# The Overlooked Impact of Botanical Pesticides on Non-Target Organisms

**DOI:** 10.3390/plants15060917

**Published:** 2026-03-16

**Authors:** Ana Paula Soares, Guilherme Julião Zocolo, Adeney de Freitas Bueno

**Affiliations:** 1 Programa de Pós-Graduação em Entomologia, Departamento de Zoologia, Centro Politécnico, Setor de Ciências Biológicas, Federal University of Paraná (UFPR), Curitiba 81531-980, Paraná, Brazil; 2 Programa de Pós-Graduação, Londrina State University (UEL), P.O. Box 10011, Londrina 86057-970, Paraná, Brazil; 3 Empresa Brasileira de Pesquisa Agropecuária, Embrapa-Soja, P.O. Box 4006, Londrina 86085-981, Paraná, Brazil

**Keywords:** bioinputs, plant-derived compounds, risk assessment, ecotoxicology, essential oils, nanotechnology

## Abstract

To better understand how botanical products affect non-target organisms, the present review focuses on the toxicity of botanical pesticides to organisms other than targeted pests, to trace a panorama on the future of sustainable agricultural models worldwide, considering the importance of ecotoxicological studies in the development of new pesticides, including botanical kinds, which are commonly recognized as essentially harmless. The review summarizes published work gathered from digital databases and highlights modern trends in pest management research and the development of novel bioinputs, including a discussion of the world’s current legislation regarding relevant agricultural innovations and field obstacles. Nanotechnology techniques are discussed as major innovations employed in the pest control field, and their employment in improving botanical pesticides is addressed and explored. In this work, we analyze the factors involved in determining the success of botanical products and their importance in the implementation of a more sustainable approach to managing crops. The results indicate a significant lack of studies focused on the effects of botanical products on non-target organisms and an increase in studies with nanoformulations.

## 1. Introduction

The world is currently undergoing a major transformation in agriculture. Sustainability has become a far more important goal than ever before, driving economies to invest in alternatives to outdated pest control techniques that expose the environment to contamination and pose risks to human health [1]. The use of non-synthetic bioinputs is becoming increasingly popular, especially when coordinated in an integrated pest management (IPM) program that allies many different techniques to diversify control strategies tailored to specific pest/crop cases [2]. Although synthetic pesticides still hold a place in agriculture, they will no longer control the market in the future, as they tend to be less adopted by farmers [3,4]. Their use is justified in strategic applications through sparse periods [5]. Biological control methods are gaining prominence because of their effectiveness in managing targeted pests while causing minimal or no adverse effects on non-target organisms (NTOs) [2], including natural enemies such as predators and parasitoids [6]. Beneficial organisms, such as biocontrol agents and pollinators, are considered NTOs that should be preserved in the environment; therefore, pest control strategies that ensure their safety should be prioritized in agriculture [7].

## 2. Types, Characteristics and Advantages of Botanical Products

Among the eco-friendly pest control tools available today, essential oil (EO)-based botanical insecticides have received increasing attention [8] because of their lower environmental persistence [9], faster degradation [10], and lower impact on NTOs [11] compared with most traditional synthetic insecticides [12]. Despite this, botanical pesticides can exert lethal and sublethal effects on both target organisms and NTOs. Lethal effects are related to the death of the organism, whereas sublethal effects can result from both acute and chronic exposures and negatively affect exposed organism populations by influencing their physiological and/or behavioral activities [13]. The types of interactions among plants and other organisms in nature are boundless. The natural interactions among these organisms provide valuable insights into mechanisms that can be further explored through scientific research. Natural insecticidal and insect-attractant plants exemplify the diverse strategies employed by nature. One reported example involves the integration of bioinsecticidal source plants with floral resources for beneficial species, resulting in dual ecosystem services that support natural enemies while providing botanical insecticides for pests [5].

**Figure 1:**
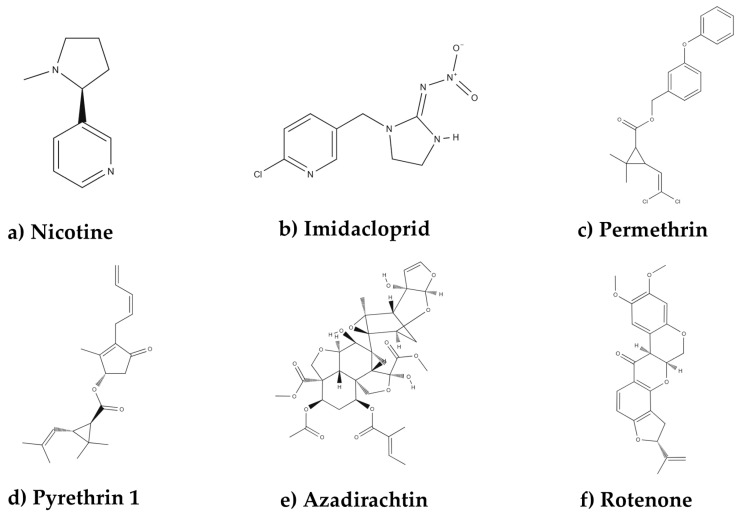
Structural molecules of different types of insecticides.

Tobacco plants produce nicotine as a chemical defense against herbivores (Figure 1a), a neurotoxin that blocks voltage-gated sodium channels in nerve axons [14]. This interesting mode of action led to the development of neonicotinoids, synthetic substances that act in a similar manner to nicotine, as exemplified by imidacloprid in Figure 1b. Similarly, pyrethroids were developed as synthetic analogues to a mixture of insecticidal compounds found naturally on the *Chrysanthemum cinerariafolium* (Trevir.) Sch. Bip. flowers. Pyrethroids, such as permethrin (Figure 1c), were developed to overcome the unstable characteristics of natural pyrethrins (Figure 1d), such as photodegradation [15,16], but their impact on NTOs remains understudied. Neem-based insecticides are produced from the seeds of *Azadirachta indica* (A. Juss), and are rich in azadirachtin (Figure 1e), a terpenoid that shows high insecticidal, repellent, anti-ovipositional and hormone-regulatory properties. Many studies analyze the effects of Neem on insects, and it has been determined to be effective in controlling at least 550 species [16]. Rotenone (Figure 1f) is also considered an important plant-derived insecticide. This substance is an isoflavonoid extracted from roots and stems of legumes of different species, such as *Tephrosia virginiana* (L.). Rotenone’s toxicity to mammals is very high and its lethal dose can be compared to DDT, a synthetic pesticide. Rotenone can act by contact and ingestion, and it can inhibit respiratory enzymes in the cell [16].

In addition to these well-characterized substances, a wide range of plant-derived products have demonstrated insecticidal activity, such as aqueous, dried, and oil-based extracts, as well as EOs [4,17]. These EOs can be used in their crude form, generally diluted, but also in blends or mixed into nano/microemulsions [18,19]. A recent study explained how most current research involving the ecotoxicology of plant-derived products focuses on commercial and industrial applications. The environmental effects of these products have received considerably less attention [17]. According to the authors’ review, over a 20-year period, only 2% of the studies on this topic were published within the environmental sciences category. This could be explained by the common misconception that all natural products tend to be harmless, even though there is substantial evidence to suggest that many plant metabolites can be toxic, posing a significant risk to the environment and to human health, albeit less than the more obvious hazards related to their synthetic counterparts [17,20].

Table 1 lists some comparisons of toxicity rates between EO-based botanical products and synthetic pyrethroids. Many studies have used *Daphnia magna* (Straus) (Anomopoda: Daphniidae) as an ecotoxicological nontarget model, considering this species’ sensitivity to synthetic pesticides at extremely low dosages, even when compared with a botanical pesticide [21]. Study models using *Eisenia fetida* (Savigny) (Opisthopora: Lumbricidae) are valuable for assessing the soil toxicity of new products. Different contamination assays using EOs of various species have shown that these botanical products are not toxic to *E. fetida*, especially when compared with the high toxicity of pyrethroids [11,22,23].

## 3. Botanical Pesticides and Their Impact on Different Kinds of Organisms

The lethal and sublethal impacts of oil-based pesticides (both EOs and botanical extracts) on different types of NTOs have been addressed in extensive reviews [6]. Current reviews evaluate how most studies (76.5%) regarding botanical product ecotoxicity are focused on the aquatic environment, while only 23.5% of all studies focus on their effects on terrestrial ecosystems. Although EOs are often considered toxic only at high concentrations, there are reports of adverse effects occurring at levels well below the limits established by international standards and regulations, affecting organisms such as microalgae, crustaceans, and fish. Therefore, it is important to emphasize that although botanical products are generally regarded as eco-friendly, they are not inherently safer than synthetic pesticides and may also pose risks to various NTOs, particularly insects, as discussed below [17,24].

### 3.1. Invertebrate Predators

Many invertebrate predators of arthropod pests play a significant role in biological control techniques. The larger size of these organisms makes them less sensitive to the effects of chemical control agents. Different studies have shown varying results regarding the selectivity of bioactive botanical compounds regarding predators, both generalist and specific types. A study has described the side effects of two Citrus EO formulations on a generalist insect predator—*Nesidiocoris tenuis* (Reuter) (Heteroptera: Miridae)—which preys on tomato pests, such as *Tuta absoluta* (Meyrick) (Lepidoptera: Gelechiidae). The formulations used were an EO-emulsion and an EO PEG-nanoparticle, developed from three different Citrus species. The authors found the mandarin EO-based insecticides in both formulations to be the most toxic against NTO, even residually, after 7 days. The mandarin EO specific composition is likely to explain the different results with the other two citrus species used in the study. Interestingly, the EO that was the least toxic to the NTO was the most toxic to *T. absoluta* eggs and larvae. The results also show that when the NTO was exposed to 3-days-old residues from all test groups, both the emulsions and the EO-nanoparticles resulted in much lower lethality rates when compared to the positive control Indoxacarb (a synthetic pesticide). Even the most lethal of the EO formulations (lemon EO-nanoparticles, with a mortality rate of 9.01% ± 3.48) was comparably less toxic than the Indoxacarb control (86.96% ± 4.07 mortality). This exemplifies an instance in which an EO can be more selective to the NTO than a synthetic counterpart [25] (Table 2).

EOs can also affect predators in a sub-lethal manner, influencing their life cycle, reproductive performance (disrupting oogenesis, vitellogenesis, maturation, and spermatocyte growth), and even their predatory abilities [6]. Voracity is a predatory parameter used to interpret the feeding ability of predators, and studies have shown that it can be positively or negatively affected by the use of EOs [3,25]. A study demonstrated that a Citrus-based biopesticide was able to increase the voracity of *N. tenuis*, when compared to a synthetic pyrethroid [26]. Another study demonstrated that *Cymbopogon citratus* (DC. Stapf) EOs and its constituents affected the respiratory rate of *Podisus nigrispinus* (Dallas) (Heteroptera: Pentatomidae), possibly by means of muscle paralysis, which affected predatory ability [27] (Table 2).

A study described how a nano-formulation enhanced the activity of natural pyrethrins against a target insect—*Aphis gossypii* (Glover) (Hemiptera: Aphididae)—while being harmless to two different non-target predators—the hemipteran *Macrolophus pygmaeus* (Rambur) (Hemiptera: Miridae) and the ladybug *Coccinella septempunctata* (L.) (Coleoptera: Coccinellidae). The results showed that pyrethrin had a superior insecticidal effect when encapsulated in the nano-formulations—when compared to commercial pyrethrin concentrates—while also being harmless to the predators. Therefore, nano-formations should be further studied and used in the development of EO-based botanical insecticides as a strategy to increase not only their efficacy against the target pest but also their safety to NTOs [28] (Table 2).

Some botanically derived substances have also been shown to attract natural predators. Studies have demonstrated that the green lacewing *Chrysoperla rufilabris* (Burmeister) (Neuroptera: Chrysopidae) and the ladybug *Harmonia axyridis* (Pallas) (Coleoptera: Coccinellidae) preferred to lay eggs on substrates treated with certain EO components compared to untreated controls [6,49,50]. It has been reported that *H. axyridis* is attracted to limonene, a substance commonly associated with alarm pheromones in aphids, which can be a chemical cue that guides *H. axyridis* to its prey [50]. Another study revealed an intriguing synergistic mechanism: the spider *Pardosa pseudoannulata* (Boesenberg and Strand) (Araneae: Lycosidae) showed no particular attraction to the EOs of *Piper nigrum* (L.) (Piperaceae) or *Litsea cubeba* (Lour.) Pers. (Lauraceae) when tested individually but was attracted to a mixture of the two. In this experiment, the EOs and their mixture were directly applied to *Nephotettix cincticeps* (Uhler) (Hemiptera: Auchenorrhyncha), a leafhopper that serves as prey for *P. pseudoannulata*. This research also clarified that the EOs of *P. nigrum* and *L. cubeba* were determined to be repellent to the pest *N. cincticeps* in the previous literature, which further verifies these oils as good candidates for bioinputs [6,29] (Table 2). This illustrates the heterogeneity of outcomes reported in EO studies and emphasizes the value of synthesizing these findings in a review to support the advancement of scientific knowledge.

### 3.2. Parasitoids

The importance of parasitoids as biological control agents has been established in the literature, referring to their success in the field to their sophisticated and uniquely adapted ways of intercepting hosts, in stark contrast to the more “crude” mode of action of predators. Despite their efficiency, parasitoids are highly vulnerable to conventional chemical pesticides, making the development of selective, low-impact alternatives an urgent priority. EOs are promising in this regard, as many formulations can be applied in a manner compatible with the release of natural enemies [3,6].

It is important to note that the selectivity of EOs to their targets and specific parasitoids depends largely on factors, such as parasitoid species, type of EO, host, parasitoid instar, and EO administration technique. In addition, many studies have compared the effects of EOs and their major components, and the results vary greatly depending on the case [6,19,25,51]. For instance, one study demonstrated how EO solutions sprayed upon parasitized eggs can affect adult emergence. The authors reported that some compounds in EOs can diffuse through the host egg chorion and affect the nervous system of the embryo, thereby interrupting its development [30]. Many studies have focused on *Trichogramma pretiosum* (Riley) (Hymenoptera: Trichogrammatidae), one of the most widely used and well-studied egg parasitoids in biological control [52]. EOs from *Hyptis marrubioides* (Epling) and *Ocimum basilicum* (L.) were demonstrated to be toxic to *Spodoptera frugiperda* (J. E. Smith) (Lepidoptera: Noctuidae) when applied topically, yet harmless to *T. pretiosum*, indicating strong potential for integration into IPM programs and reinforcing previous evidence of *H. marrubioides* EO effectiveness against *S. frugiperda* via ingestion essays [31] (Table 2).

A meta-analysis review on the effects of different botanical insecticides (extracted from plants, such as *Azadirachta indica*, *Calceolaria andina*, *Cymbopogon schoenanthus*, *Lantana camara*, and *Schinus molle*, among others) on hymenopteran parasitoids found that these products often increased mortality and reduced parasitism and emergence. However, the magnitude of these effects can vary with the plant source, parasitoid family, and exposure type. For example, parasitoids in the families Scelionidae and Ichneumonidae were far less affected than those in the other families examined, likely due to differences in detoxification capacity. Negative effects perceived on parasitoid survival were most pronounced in ingestion and residual assays, whereas topical exposure resulted in fewer detrimental outcomes. These findings highlight the need for case-specific assessments to identify application strategies that maximize pest control while protecting biological control agents, establishing the best strategy to use those botanical insecticides efficiently and cautiously [51].

A study evaluated the potential of *C. citratus* (DC.) Stapf EO for controlling *S. frugiperda* and its selectivity towards the egg parasitoids *T. pretiosum* and *Telenomus remus* (Nixon) (Hymenoptera: Scelionidae). Fumigation assays showed high mortality rates for *S. frugiperda* larvae and *T. pretiosum* adults (over 90%), whereas *T. remus* exhibited much lower mortality (12–38%), highlighting how EOs can elicit species-specific responses and the importance of selecting compatible parasitoid–EO combinations in IPM [32] (Table 2).

Similarly, a study assessed the toxicity of EOs from three *Baccharis* sp. species against *S. frugiperda*, through topical and ingestion exposure, and the EOs’ selectivity to *T. remus*, across different developmental stages (Table 2). Their findings showed pronounced chemical and biological variations among species of *Baccharis* sp.: the oils induced neurotoxicity and lipid peroxidation in *S. frugiperda*, while two Baccharis EOs caused no mortality in *T. remus* eggs or pupae, despite exhibiting strong repellency. These studies demonstrate how chemical divergence among closely related plant species can configure EO selectivity, reinforcing the potential of botanical bioinputs as sustainable alternatives to synthetic pesticides when carefully matched to compatible NTOs [33].

A study tested the selectivity of three EOs—*Lippia origanoides* (Kunth), *Cymbopogon winterianus* (Jowitt), and *C. citratus* to the egg parasitoid *T. pretiosum*. All oils tested showed selectivity to *T. pretiosum* at different concentrations, with *L. origanoides* being considered the least harmful of the three, and the other oils being considered slightly harmful (Table 2). However, this work also demonstrated that spraying *S. frugiperda* eggs with higher oil concentrations reduced parasitism rates and adult emergence of *T. pretiosum*, in an inversely proportional manner. Therefore, the negative side effects of NTOs must be evaluated in relation to the application rate, rather than being attributed solely to the EO per se. The authors clarify that the low residual power observed in EOs dispersed in fields greatly reduces their period of insecticidal activity, and aligning the release of biological control agents such as parasitoids to periods either before or after spraying crops with biological products can be critical in determining the success of an IPM program [30].

### 3.3. Insect Pollinators

Many studies on pesticide impacts on pollinators have relied heavily on honeybees—*Apis mellifera* (L.)—as study models, a justified choice given their ecological value. However, Brazil alone hosts 2000 catalogued native bee species [53], making it essential to expand ecotoxicological studies beyond *Apis mellifera* (Hymenoptera: Apidae); by Giunti Pollinators play a crucial role in crop production, and several cases demonstrate that EO considered safe for crops may still be harmful to these insects [6]. Oils from *Corymbia citriodora* (Hook.) K.D.Hill & L.A.S. Johnson and *Artemisia annua* (L.), for example, have shown strong insecticidal activity to pests, but also significant toxicity to the stingless pollinator bee *Tetragonisca angustula* (Latreille) (Hymenoptera: Apidae, Meliponinae) (Table 2). Even so, these products aren’t disqualified from use in IPM programs; adjusting application timings—such as spraying during non-flowering stages—can greatly reduce pollinator exposure. By understanding the life cycle of different links in the ecosystem chain, we can better employ accurate methods to each of the plant’s life stages [34,35] (Table 2).

Conversely, several EOs are shown to be innocuous or even repellent to beneficial bees. *Lippia sidoides* (Cham.) EO and its major compounds caused low toxicity and no motor impairment in contact essays with *Nannotrigona testaceicornis* (Lepeletier) (Hymenoptera: Apidae, Meliponinae), despite repelling the species [36] (Table 2). Studying such selective interactions can aid the preservation of pollinator communities, since it can boost the development of products particularly tailored to very specific cases, granting better manipulation of the crop systems in favor of the maintenance of beneficial fauna and effective pest control [1,3].

*Apis mellifera* comprises 80% of all insect pollinators in the world and poses economic importance for the honey and beeswax market. They are sensitive bioindicators of pollutants, heavy metals and pesticides [6,54]. A study shows that this species can be affected by *Lippia gracilis* (Schauer) EO and its isolated major compounds which also affected another pollinator, *Polybia micans* (Ducke) (Hymenoptera: Vespidae) [37] (Table 2).

Apart from lethal toxicity, many studies also investigated sublethal effects botanical insecticides can cause in these organisms, such as interference on movement and flight patterns. *A. mellifera* bees walking activity was negatively affected by its exposure to eucalyptus and Neem EO [55]. It can be speculated that botanical pesticides such as the ones based on EOs can impact the physiology of bees by affecting the nervous system. Low concentrations of EOs and its single compounds have been shown to increase acetylcholinesterase and glutathione S-transferase activities [56].

In contrast, studies also demonstrate how certain EOs can be considered safe to different species of bees. The EO of *Persea venosa* (Nees & Mart.) was effective against *Dysdercus peruvianus* (Guerin-Meneville) (Hemiptera: Pyrrhocoridae), while being harmless to both *A. mellifera* and *Partamona helleri* (Frese) (Hymenoptera: Apidae, Meliponini)—a bee species native to Brazil [38] (Table 2). A plant extract from *Mammea siamensis* (T. Anders) demonstrated potential as a botanical pesticide: the isolated major compound was tested against different non-target organisms, and *A. mellifera* wasn’t affected, while other organisms were [39] (Table 2).

Furthermore, some works have also focused on the effects of isolated volatile substance like methyl benzoate, which is found in many different plants. Studies have pointed to this substance’s potential repellent and/or attractant activity depending on insect species, its effect on insect behavior, and its documented toxicity to certain insects. Not only was this compound highly effective against *S. frugiperda*, but it was also non-toxic to both a pollinator—*Bombus terrestris* (L.) (Hymenoptera: Apidae)—and two natural predators (*C. septempunctata* and *H. axyridis*) [40] (Table 2).

### 3.4. Aquatic Organisms

The majority of studies focusing on the potential toxicity of plant derived bioinsecticides on NTOs are directed towards aquatic invertebrates. *Daphnia magna* is a widely used toxicological model [57]. While some Eos—such as the one from *Pimpinella anisum* (L.) show strong larvicidal activity against the mosquito *Culex quinquefasciatus* (Say) (Diptera: Culicidae), they can also cause high *D. magna* mortality at elevated concentrations, suggesting the need for adjusted doses [41] (Table 2).

There is a scarcity of research evaluating impacts on microalgae, despite their foundational role in aquatic food chains [17]. Toxicity data for nanoparticle-based botanicals are inconsistent and varied: for example, studies with silver-nanoparticle plant extracts show acute toxicity to *D. magna* and other taxa (such as fish, crustaceans and phytoplankton), while other EO-based products appear to be non-toxic to *D. magna* [42,43] (Table 2).

Other NTOs also warrant attention. Pyrethrum-loaded nanoparticles caused hematological alterations and DNA damage in tadpoles of *Lithobates catesbeianus* (Dubois) (Anura: Ranidae) [44] (Table 2). Conversely, compounds from *Schinus terebinthifolius* (Raddi) proved effective against *Culex pipiens* (L.) (Diptera: Culicidae) while remaining safe for earthworms and the fish species *Gambusia affinis* (Baird and Girard) (Cyprinodontiformes: Poeciliidae) [58] (Table 2).

Given the critical ecological roles of both freshwater and marine ecosystems [58], expanding toxicological studies across different aquatic groups is essential to ensure the safe use of botanical pesticides and maintain ecosystem integrity and biodiversity.

### 3.5. Soil Invertebrates, Microorganisms and Enzymes

Soil contamination by botanical pesticides is also an important dimension of ecological safety, since many bioindicator organisms live in the soil and impact nutrient cycling and crop productivity [6]. Earthworms, especially *Eisenia fetida*, are widely used in ecotoxicological assays, and most studies report that EOs pose little to no risk to this species [11]. Even an EO nanoformulation did not seem to affect *E. fetida*, despite increasing target bioactivity when compared to a crude EO [22] (Table 2).

A study demonstrated that a commercial EO-based biopesticide was non-toxic to *E. fetida*, the collembolan *Folsomia candida* (Willem) (Entomobryomorpha: Isotomidae) and the predatory mite *Hypoaspis aculeifer* (Canestrini) (Acari: Laelapidae) [45] (Table 2). Likewise, another study regarding 18 EOs showed that most of them only showed moderate toxicity against *Proisotoma minuta* (Tullberg) (Entomobryomorpha: Isotomidae), though three of those EOs were strongly toxic [46] (Table 2). Notably, eucalyptus EO, that was not toxic against *P. minuta*, had been previously shown by the same authors to be highly effective against *Sitophilus oryzae* (L.) (Coleoptera: Curculionidae), reinforcing how EO toxicity can be species-specific.

A different study found that the main compound of *M. siamensis* was harmless to the earthworm *Pheretima peguana* (Rosa) (Opisthopora: Megascolecidae), whereas the synthetic insecticide methomyl was 50 times more toxic [39] (Table 2). The plant volatile “methyl benzoate” showed limited soil accumulation and posed no risk to *E. fetida* [40] (Table 2). Compounds from the Brazilian plant *Schinus terebinthifolius* were also effective against *Culex pipiens* while remaining safe for *E. fetida*: a study tested the EO, its nanoemulsion and isolated monoterpenes. The three compounds showed mosquitocidal, repellent and acetylcholinesterase inhibitory activities against the target insect, and were safe toward *E. fetida*. The EO presented more potent bioactivity than each individual isolated terpene, and among all treatments, the nanoemulsion showed the strongest bioactivity against *C. pipiens* [22] (Table 2).

Soil nematodes can be important bioindicators and are often overlooked in pesticide evaluations [59]. These organisms can also show resilience to certain botanical products. Basil EO (*O. basilicum*) applied along with chitosan as a seed coating did not disrupt nematode taxonomic diversity, even at high concentrations, while still suppressing Fusarium sp. infections on seeds [24] (Table 2).

Microbial communities also play central roles in soil health, yet their interactions with botanical pesticides remain understudied. A study investigated the effects of 23 different EOs on three beneficial bacteria species commonly used as bioinputs in agriculture (in the form of biostimulants and biocontrol antagonists) and showed that most of the tested EOs and were compatible with all the beneficial bacteria, though a few of those EOs (such as oregano oil) exerted inhibitory activity against the bacteria species tested [47] (Table 2). These insights highlight the need to examine oil composition when predicting microbial selectivity. Another study showed that Citrus EO formulations did not disrupt microbial abundance or enzymatic activity in soil and were shown to not induce oxidative stress in plants [25], unlike many synthetic pesticides [60]. Some EOs can even be used as carbon sources for soil microbiota [61].

Overall, current evidence suggests that botanical products generally exert a limited impact on soil biota, although species-specific responses exist and must be considered. Since soil ecosystems are complex and biologically diverse, conducting targeted ecotoxicological assessments remain essential steps before choosing botanical pesticides for agricultural systems.

### 3.6. Mammalians

Not many studies focus on the effects of EOs on mammalians, but it is important to explore that area, since mammals—such as humans and other animals—can be indirectly affected by any pesticide, including EO-based products [17]. A study evaluated the effects of a larvicidal nanoemulsion based on the EO of *Aeollanthus suaveolens* (Mart. ex Spreng) against *Aedes aegypti* (L.) (Diptera: Culicidae), and its potential toxicity against non-target mammals, using mice as models. The authors found that the EO nanoemulsion was effective against the insect target, especially in concentrations over 60 μg/mL. The nanoemulsion did not induce significant changes in weight of the heart, liver, lung and kidneys of mice, and the histopathological tests reveal no changes in the heart of the treated animals, but some alterations were observed in the lungs, kidneys and liver. The authors highlight the fact that nanoemulsions can have a particularly pronounced effect on histological tissues, because the small size of particles used in the emulsion favors a rapid absorption by animal tissues and fast metabolism, which corroborate to a toxic effect [48] (Table 2).

A nanoemulsion of the *Croton linearis* (Jacq.) EO was tested for its larvicidal activity and toxicity in NTOs. The results demonstrated the nanoemulsion to have a more potent larvicidal effect than the crude EO. They tested for cytotoxicity on non-neoplastic human lung fibroblasts and for hemolytic effects on murine erythrocytes, as well as acute oral toxicity on rats. As results, they found no hemolytic effect, nor cytotoxicity. The animals in the acute toxicity test presented no organ abnormalities and no abnormal behaviors, which suggests that the nanoemulsion can be classified as a nontoxic product and safely used in the field [19] (Table 2).

More studies focusing on mammalian animals/cells/tissues need to be developed to further investigate a vastness of EOs, their main compounds, nanoemulsions and blends. Factors like formulation type, composition, chemical properties and modes of action can greatly influence the toxicity of these products. That is essential to avoid mammal exposure to these substances, and to popularize the use of EOs as matching competitor to synthetic pesticides.

## 4. An Evolutionary and Molecular Perspective on Possible Targets of Botanical Products

EOs are classified as “phytocomplexes”, i.e., a blend of different types of substances, that may include terpenes (generally constituting the majority), phenylpropanoids, aldehydes, alcohols, esters, ketones, alkanes and polyacetylenes. EOs are stored in different parts of the plant (like roots, flowers, leaves, seeds, fruits), thus, requiring different extraction methods. Among some of the most common are hydrodistillation, steam distillation and cold pressing. Each type of extraction technique has advantages and disadvantages in terms of yield, efficiency, and substance preservation [6]. The efficacy of EOs against pests varies greatly within the same genus because different chemotypes are common. This also means that the effects on NTOs may vary [6]. EOs are products of plant secondary metabolism, and their synthesis is influenced by multiple environmental factors, such as nutrient availability, irrigation, sunlight exposure, and genetic determinants. Closely related plant species may share similar gene expression patterns and biosynthetic enzymes involved in metabolic pathways, conferring taxonomic relevance to certain phytochemicals. However, the occurrence of these compounds is often associated with specific adaptive traits within a given phylogenetic framework [62].

Similar to other secondary metabolites, EOs play important roles in interspecific interactions between plants and other organisms, extending beyond mere adaptation to environmental conditions. Certain transcription factors, like the R2R3 MYB domain, have been linked to plant’s peripheral processes and thus, the secondary metabolism [63]. This group of transcription factors is known to regulate plant’s resistance to different pathogens and diseases [64].

The many phytochemicals found in plants are directly related to their evolutionary adaptations to environmental stress and the establishment of different ecological interactions, such as those with predators and pollinators. In recent years, the focus on research into gene sequencing has been able to elucidate many mechanisms behind the production of these compounds; by describing the evolution of metabolic enzymes and the biosynthetic pathways they play a part in [63].

Maeda and Fernie [65] postulated that the immense plant chemical *repertoire* observed today is partially explained by the transformation of core metabolic enzymes (the oldest kinds, prevalent since the beginning of the history of plants). This mechanism is facilitated by gene duplication, which the authors specified was very prevalent in the plant domain [65]. This explains why secondary metabolites are estimated to number between 200,000 and 1,000,000, in comparison to only 1000 primary metabolites: the chemical diversity found in secondary metabolites derives from millions of years of evolutionary adaptations through mutations and natural selection [66].

Plant-derived products, such as EOs and extracts, can act selectively on pests owing to a myriad of reasons, including evolutionary perspectives and physiological factors, with evidence sustaining the fact that these substances have lower risks to beneficial NTOs. There can be many differential biochemical reactions, such as a much higher binding affinity of bioactive compounds to a target organism’s key receptors, such as GABA and octopamine receptors. Some examples in the literature indicate that alternative products based on the EOs of *Syzygium aromaticum* (L.) Merr & L. M. Perry, *Ocotea indecora* (Schott) Mez and *Pectis brevipedunculata* (Gardner) Sch.Bip. target specific receptors on pest targets, while they do not affect the NTOs, for example, by being innocuous to the predatory abilities of beneficial insects or to the feeding of pollinators, which is a good indicator for an environmentally friendly pesticide [5,67,68,69].

Many insecticides trap voltage-gated ion channels and cause paralysis or tremor effects, such as TRP channels. Some variants of these channels are absent in honeybees but are present in many insect pests. This may explain why some EOs affect target pests more than bees, for example. Another interesting case discussed by the authors relates to detoxification enzymes: studies have shown that certain EOs selectively inhibit acetylcholinesterase (AChE, an enzyme that catalyzes the breakdown of acetylcholine, an important neurotransmitter) activity in target insects and do not appear to impair AChE activity in other non-target invertebrates [6].

Other interesting mechanisms are plant-derived substances actions on insect growth systems. Some plant components can cause disruption in insect development and growth, affecting molting and metamorphosis. Some targets include the methoprene-tolerant receptor, the juvenile hormone primary receptor, and the ecdysone receptor [6]. Studies demonstrated a developmental delay in *Dysdercus peruvianus* exposed to the EO of *Persea venosa*, while the same EO (and its isolated main compound, beta-caryophyllene) showed selectivity to pollinator bees, with a high survival rate [38].

These studies demonstrate different ways in which plant-derived substances can exert selective effects on target organisms while having little to no effect on beneficial organisms.

## 5. Facing Challenges in the Future of Botanical Insecticides Market: Regulatory Barriers

Many of the modern-day barriers faced by botanical insecticides were common nearly three decades ago: resource availability, standardization/quality control, and regulatory requirements for registration purposes and legality of use. These factors directly impact the availability of botanical insecticides on the market [70]. Considering regulatory requirements, all studies involving genetic information from organisms in the Brazilian national territory are subjected to the law No. 13.123/2015, (which encompasses plants and their bioproducts, among other organisms), regulating access to genetic heritage, protection and access to associated traditional knowledge, and for benefit sharing for conservation and sustainable use of biodiversity. This law regulates the access to the genetic property of native plants and other organisms commonly used in the research field. The law also implements the need to register activities involving access to genetic material into a nation-wide database through the SisGen system, and the submission of notifications before commercialization of a final product or reproductive material [71].

In Brazil, the Decree No. 10.375 of 26 May 2020, establishes a program of strategic actions that focus on a direct investment of financial resources, and promotion of science, technology and innovation fields. The “National Bioinputs program” encourages the widespread adoption of biological inputs in agriculture, developing a more sustainable approach to the modern agricultural scenario. These so-called “bioinputs” encompass a range of products such as seeds, fertilizers and inoculants, nutrition products for livestock and plants, botanical extracts, biological control organisms for field dispersal, veterinary vaccines, and various other technologies [72]. The establishment of this nationwide program has been essential to spread new technologies in both the national and the international market, reaching even small-scale producers, by bringing innovative products and technical knowledge to family farms focused on organic products, for example, even though this process is still moving at a slow pace, and the program still has to expand bioinputs accessibility and diversity to these very important groups [73].

Considering how botanical pesticides are generally perceived as innocuous and environmentally safe, it is extremely important that national laws and regulations enforce the rigor of academic research focusing on the development and application of new botanical products, to ensure that the impact of these products on non-target organisms is as minimal as possible. The employment of strict nationwide regulations by health surveillance organs. According to ANVISA’s (National Health Surveillance Agency) online domain, it is established that IBAMA (Brazilian Institute of Environment and Renewable Natural Resources), MAPA (Ministry of Agriculture and Livestock) and ANVISA work together to guarantee the safety of pesticides in national territory, and ANVISA is directly involved in performing the toxicological assessment of registered products, determining safe guidelines and proper use conditions. Botanical pesticides are fully included in the scope of this legislation, and the proper regulation of their use is imperative in assuring that NTOs are safe from possible harm. These same institutions are responsible for guaranteeing that imported products are either allowed or banned on the national territory.

A recent meta-analysis revealed that Asia concentrates the majority of scientific production and research on phytochemicals, with the greatest amount of research being published in China. Brazil is the leading country in the Americas in this field of knowledge. European and African countries are underrepresented, which symbolizes a research gap in those regions. The authors’ database did not show any studies originating from Oceania [74]. The extent to which a country’s scientific community prioritizes and focuses efforts on topics such as pesticide health regulation can substantially influence the development of new, modern legislations, particularly when pesticide use is closely tied to economic factors, including agricultural exports and commodity markets—which is the case in Brazil.

The global scenario regarding the regulation of pesticides varies greatly. In India, laws such as The Insecticides Act of 1968 and The Insecticides Rules of 1971 represent a barrier in the development of botanical pesticides, as this legislation was originally developed for the regulation of chemical pesticides and subsequently extrapolated for modern pesticides. Considering the disparate nature of these types of products, these laws can be archaic and slow down the manufacturing and sale of botanical insecticides. Experts in the scientific community have postulated that biologically produced microbial, botanical, and pheromonic biopesticides should be considered subjects of separate, dedicated policies in India. The authors compared India’s regulatory framework with Canadian legislation, noting that in Canada, the development and use of botanical products (and other biological pesticides) fall under The Pest Control Act of 2002, which is comparatively more structured and provides a more targeted approach to the distinct nature of modern biological pesticides, evaluating them separately from conventional synthetic pesticides [75].

Many studies have highlighted that botanical pesticides being evaluated under the same regulatory framework as synthetic pesticides is a common problem worldwide. This can lead to several issues regarding the popularization of botanical pesticides, including increased registration costs and tests. This is the case in the European Union, in which biopesticides also fall under the same laws as synthetic pesticides. The United States are, along with Japan, home to the main major industries in the world, which amplifies testing and registration fees. Unlike most countries, the United States have flexible laws regarding biopesticides, since the Environmental Protection Agency has a special category for biochemical pesticides. Authors have also linked inadequate risk assessment regarding indiscriminate pesticide use in developing countries to cases of poisoning and environmental contamination. It is also noted that a major selling point for botanical products in developing nations is the fact that many tropical countries are rich in biodiversity and native plants are readily available and have low cost [76].

## 6. Developing Technologies to Boost Botanical Insecticides Competitivity on the Market

The national organic market is growing increasingly, reflecting a global shift toward sustainable agricultural practices grounded in “green chemistry”. Organic farming systems in Brazil represent a disruptive alternative to conventional agriculture, emphasizing ecological complexity, functional biodiversity, sustainable management, and integration with natural elements [77]. These principles support healthier production systems and more balanced interactions with the environment, and the increasing demand for sustainable bioinputs is now extending well beyond the organic sector.

Only a few plant species—such as Neem and pyrethrum—have been truly extensively studied for their insecticidal properties, while countless other species remain unexplored despite their potential to yield new bioactive compounds for crop protection. The development of botanical pesticides relies heavily on the study of these compounds, and the understanding of their biosynthesis and modes of action [14]. Brazil is the most biodiverse country in the world, home to 15–25% of all plant species, with a very high rate of biological endemism [77]. This fact, allied to national politics encouraging the cataloguing of plants and the sponsorship of more researchers in the field of bioinputs development, constitute a major and pivotal interest area in the development of a more sustainable future.

Commercial EO-based products can contain one or more types of EO, a blended mixture of many EOs, or a blend of synthetic substances, such as terpenoids (which can be naturally found on EOs). The synthetic production of bioactive compounds can be more economically viable when compared to the difficulties involved in extracting large amounts of EO. Requiem Prime^®^ is an example of a current insecticide product developed synthetically from a terpenoid blend [78].

The same qualities that make EOs good candidates for sustainable biopesticides are what limit their use on the market. For example, the low toxicity and high biodegradability of these compounds are favorable from an environmental perspective; however, this translates into products with short shelf lives [6]. To overcome the problems associated with the intrinsic properties of EOs, such as volatility and rapid degradation in nature, modern approaches, such as the use of nanotechnology, have become extremely popular.

A great concern about the use of EOs as pesticides in agricultural systems is the large amount of biomass needed to yield a small amount of oil, and the frequent disparities in productivity depending on many factors. Some authors regard the differences in EO productivity to factors such as climate, soil composition, geographic location, seasonal variations, part of the plant used, plant age, cycle stage and harvest period. The type of extraction and drying of the material can influence productivity. Thus, to determine if an EO is economically viable—for example, as a botanical pesticide—it’s necessary to assess the number of harvests per year, how much final product is yielded and the duration of each harvest period [48].

Recently, nanotechnology techniques have been employed to enhance the properties of natural products, including botanically derived compounds. Nanotechnology is a valuable tool to improve the quality of natural products by extending their shelf life and regulating their release periods after application, and can offer a significant impact on the value of a potential product. EOs in their crude form are quickly degraded in nature, and nanotechnology techniques can help elaborate products with more stability and market value, diminishing the impacts of photodegradation and volatilization of metabolites and enhancing field performance [79].

There are many examples of nanotechnology-based studies reinforcing the activity of EOs and their metabolites with carrier systems, like nanoencapsulation and microencapsulation studies [80]. A study employed the method of high-energy emulsification of an EO and showed great results in controlling *Planococcus citri* (Risso) (Hemiptera: Pseudococcidae) increasing the oil’s efficacy, while also being harmless to non-target honeybees [80]. In a study, a Neem gum nanoformulation was found to be successful against two species of moth larvae and pupae [81]. Another work provides a very compelling example of how nanotechnology can help mitigate unintended toxicity of bioactive compounds toward NTOs: Neem EO was previously shown to induce genotoxic effects on NTOs like *Allium cepa* L., and by interfering with the physiology of *Caernorhabditis elegans* Maupas (Rhabditida: Rhabditidae), a soil nematode. A formulation designed by the authors consisted of the Neem EO being nanoencapsulated into zein-based polymeric nanoparticles. Tests developed with this substance showed that in comparison to pure EO, the nanoformulation demonstrated less genotoxic effects in *A. cepa* (decreasing chromosomal aberrations and mitotic index, while the EO increased aberrations greatly) and also eliminated the sublethal physiological stress effects in *C. elegans* that are observed when these NTOs are exposed to pure Neem EO. These results show how nanotechnology can improve a botanical product by overcoming its practical field limitations [82].

Many studies focus on the enhancement of pure EOs through nanoformulations. In one case, a nanoemulsion from *Croton linearis* (Jacq.) that was more effective in controlling mosquito larvae than the pure EO [19]. A study tested a pure EO, its isolated compounds and an EO nanoemulsion, and found that the nanoemulsion yielded the best results against a target pest, while still being harmless to NTOs [22]. While these examples are promising, this doesn’t mean nanoparticles are perfectly clean delivery systems, since nanoparticles bioaccumulation in aquatic and terrestrial systems is a topic that still requires attention [83].

The success of botanical pesticides and their market competitiveness rely on their ability to rise above other consolidated products, by offering minimal ecological impact and environmental disruption while sustaining effective pest control results and supporting the conservation of NTOs. EOs can potentially offer specificity on NTOs’ selectivity, allowing finer discrimination among beneficial species and pests, and that is a highly valuable feature in today’s sustainability-oriented market. Ensuring the safety of NTOs is a foundation of environmentally responsible pest management programs.

However, it’s important to stress that EOs and other botanical products safety cannot be assumed; they must be rigorously evaluated by laboratory and field studies, since selectivity to botanical products like EOs varies not only among species, but also across developmental stages and exposure methods. The efficacy of botanical pesticides mustn’t compromise the survival of NTOs and the key ecological services they provide, like the natural regulation of pest populations. The selective toxicity of botanical pesticides is a decisive advantage in a sustainable agriculture model, and EO-based products arise as very promising tools for IPM programs, allowing productivity without sacrificing biodiversity, nor health.

## 7. Conclusions

The development of botanical pesticides involves the cultivation and extraction of plant biomass, and that must be taken into consideration when evaluating if a product is economically viable. Also, the assumption that natural products are inherently benign is a misconception which needs to be addressed and demystified, since it prevents science from focusing on natural products’ potential hazards, resulting in a scarcity of literature in this field, especially when it comes to non-target organisms other than mammals. Despite, in general, botanical insecticides being less toxic than their synthetic chemical counterparts, non-target effect must be studied and determined case by case. Therefore, there is a dire need for more research efforts in determining health risks associated with botanical products, even if only to exclude potential dangers. In summary, there is more to the future of sustainable agriculture than the discovery of new bioactive compounds. The current biotechnological and chemical innovations in the agricultural sciences field need to be coupled with an environmentally healthy perspective that expands beyond economic-restricted views and encompasses sustainable trends, and the study of ecotoxicological impacts of bioinputs on NTOs is a reflection of that shift into the future.

## Figures and Tables

**Table 1:** Examples of different botanical product toxicity rates compared with synthetic pesticides, such as α-cypermethrin, and their effects on NTOs.

Non-Target Organism	Type of Test	Evaluation Time	Negative Control	EO Mean (Concentration): Mean Mortality (%)	Positive Control/Synthetic Pesticide Concentration: Mean Mortality (%)	References
*Eisenia fetida*	Artificial soil contamination assay	5 days	Distilled water: 0.0 ± 0.0			[11]
				*Ocimum gratissimum* EO (200 mg kg^−1^): 0.0	α-cypermethrin (20.0 mg kg^−1^): 95.5	
				*O. gratissimum* aqueous extract 200 mg kg^−1^): 0.0	α-cypermethrin 10.0 mg kg^−1^): 77.3	
				*O. gratissimum* ethanolic extract 200 mg kg^−1^): 0.0	-	
	
*Eisenia fetida*	Artificial soil contamination assay	5 days	Distilled water: 0.0 ± 0.0			[22]
				*Schinus therebinthifolius* EO (100 mg kg^−1^): 0.0	α-cypermethrin (20.0 mg kg^−1^): 89.6	
				*S. therebinthifolius* NE (100 mg kg^−1^): 0.0	α-cypermethrin (10.0 mg kg^−1^): 55.3	
	
	
*Eisenia fetida*	Artificial soil contamination assay	7 days	Distilled water + Tween 80: 0.0 ± 0.0	*Thymus daenensis* EO (200 mg kg^−1^): 5.0	-	[23]
				*Satureja sahendica* EO (200 mg kg^−1^): 0.0		
				*Satureja khuzestanica* EO (200 mg kg^−1^): 0.0	α-cypermethrin (0.1 mg kg^−1^): 85.0	
				*Satureja rechingeri* EO (200 mg kg^−1^): 0.0		
				*Oliveria decumbens* EO (200 mg kg^−1^): 0.0		
*Daphnia magna*	Acute aquatic toxicity test	48 h	Distilled water + DMSO: 0.0 ± 0.0	*Peumus boldus* EO (96.2 mg·L^−1^): 66.2	α-cypermethrin (0.025 mg L^−1^): 100.0	[21]

Abbreviations: EO = essential oil; NE = nanoemulsion.

**Table 2:** Different plant-derived substances tested on target and non-target organisms.

Substance Tested	Target Species	Toxicity Impact to Non-Targets	Non-Target Species/Enzymes/Cells	Toxicity Impact to Non-Targets	References ^1^
*Croton linearis* EO nanoemulsion	*Aedes aegypti*	Significant	Murine erythrocytes; non-neoplastic human lung fibroblasts	Negligible	[19]
*Ocimum basilicum* EO formulations	*_* ^2^	_	Soil nematode communities	Negligible	[24]
Citrus EOs NE and NP (lemon, mandarin and sweet orange)	*Tuta absoluta*	Significant (Only for mandarin EO)	*Nesidiocoris tenuis*; soil and plant enzymes	Negligible	[25]
Prev-Am^®^ (Citrus peel oil)	*Tuta absoluta*	Significant	*Nesidiocoris tenuis*	Negligible	[26]
*Cymbopogon* *citratus* EO and its major compounds	_	_	*Podisus nigrispinus*	Negligible	[27]
Pyrethrin nanoformulations	*Aphis gossypii*	Significant	*Coccinella septempunctata*; *Macrolophus pygmaeus*	Negligible	[28]
*Piper nigrum* and *Litsea cubeba* EOs and their mixture	*_*	_	*Pardosa pseudoannulata*	Significant to *P. nigrum* EO and EO mixture, negligible for *L. cubeba* EO	[29]
*Lippia origanoides*, *Cymbopogon winterianus* and *Cymbopogon citratus* EOs	_	_	*Trichogramma pretiosum*	Negligible	[30]
*Hyptis marrubioides* and *Ocimum basilicum* EOs	*Spodoptera frugiperda*	Significant	*Trichogramma pretiosum*	Negligible	[31]
*Cymbopogon citratus* EO	*Spodoptera frugiperda*	Significant	*Trichogramma pretiosum*; *Telenomus remus*	Significant for *T. pretiosum*, negligible for *T. remus*	[32]
EOs from various *Baccharis* species	*Spodoptera frugiperda*	Significant	*Telenomus remus*	Negligible (But some repellency effects observed)	[33]
EOs from various *Corymbia* and *Eucalyptus* species	*Ascia monuste*	Significant for *Corymbia citriodora*	*Solenopsis saevissima*; *Tetragonisca angustula*	Significant for *T. angustula*, negligible for *S. saevissima*	[34]
EOs from various *Artemisia* species	*Diaphania hyalinata*	Significant	*Solenopsis saevissima*; *Tetragonisca angustula*	Negligible	[35]
*Lippia sidoides* EO and major compounds	*_*	_	*Nannotrigona. testaceicornis*	Negligible	[36]
*Lippia gracilis* EO and major compounds	*Diaphania hyalinata*	Significant	*Apis mellifera*; *Polybia micans*	Significant	[37]
*Persea venosa* EO	*Dysdercus peruvianus*	Significant	*Apis mellifera*; *Partamona helleri*	Negligible	[38]
*Mammea simensis* extract and bioactive compounds	*Plutella xylostella*	Significant	*Apis mellifera*; *Pheretima peguana*; *Oreochromis niloticus*	Significant to *O. niloticus*, negligent to *A. mellifera* and *P. peguana*	[39]
Methyl benzoate (isolated plant volatile)	*Spodoptera frugiperda*	Significant	*Bombus terrestris*; *Coccinela eptempunctata*; *Harmonia axyridis*	Negligible	[40]
*Pimpinella anisum* EO	*Culex quinquefasciatus*	Significant	*Daphnia magna*	Significant (In certain concentrations and exposure time)	[41]
*Alcea rosea* biosynthesized silver nanoparticles	*_*	_	*Chlorella vulgaris*; *Daphnia magna*; *Danio rerio*	Significant	[42]
*Mentha piperita* and *Cymbopogon martinii* EOs and their polymeric nanoparticles	*Sitophilus oryzae*; *Lasioderma serricorne*; *Culex pipiens pipiens*	Significant	*Tenebrio molitor*; *Blaptica dubia*; *Artemia salina*	Significant for *A. salina*, negligent for *T. molitor* and *B. dubia*	[43]
Pyrethrum extract and solid lipid nanoparticles loaded with pyrethrum	*_*	_	*Lithobates catesbeianus*	Significant	[44]
*Schinus terebinthifolius* EO, its nanoemulsion and isolated compounds	*Culex pipiens*	Significant	*Gambusia affinis*; *Eisenia fetida*	Negligible	[22]
Novaluron-based insecticides and vegetable oil adjuvant	*_*	_	*Eisenia andrei*; *Folsomia candida*; *Hypoaspis aculeifer*	Significant to one of the formulations	[45]
18 different EOs	*_*	_	*Proisotoma minuta*	Significant to citronella, petitgrain and thyme EOs	[46]
23 different EOs and major compounds	*_*	_	*Bacillus cereus*; *Bacillus velezensis*; *Priestia megaterium*	Partially significant to some EOs and bacteria species	[47]
*Aeollanthus suaveolens* nanoemulsion	*Aedes aegypti*	Significant	*Mus musculus*	Negligible	[48]

Abbreviations: EO: essential oil, NE: nanoemulsion, NP: nanoparticles. ^1^ Listed in order of citation. ^2^ No specific test was conducted in the study.

## Data Availability

All data generated or analyzed during this study are included in this published article.

## Abbreviations

The following abbreviations are used in this manuscript:

EO	Essential Oil
CNPq	Conselho Nacional de Desenvolvimento Científico e Pesquisa
NTO	Non-Target Organism
IPM	Integrated Pest Management
EMBRAPA	Empresa Brasileira de Pesquisa Agropecuária
MAPA	Ministério da Agricultura e Pecuária
ANVISA	Agência Nacional de Vigilância Sanitária
IBAMA	Instituto Brasileiro do Meio Ambiente e dos Recursos Naturais Renováveis
